# Distinct CFTR Mutation Spectrum and Atypical Clinical Presentations in Chinese Patients with Cystic Fibrosis

**DOI:** 10.3390/ijms27062770

**Published:** 2026-03-18

**Authors:** Zixin Wang, Guizhi Zuo, Ye Shi, Yinghao Zhao, Xue Fan, Xia Hou, Qingtian Wu

**Affiliations:** School of Basic Medicine, Jiamusi University, Jiamusi 154007, China; wzx3169624335@163.com (Z.W.); 15233499116@163.com (G.Z.); 13938183711@163.com (Y.S.); 15636999713@163.com (Y.Z.); 15505436929@163.com (X.F.)

**Keywords:** cystic fibrosis, *CFTR* mutations, Chinese population, mutation spectrum

## Abstract

Cystic fibrosis (CF) is an autosomal recessive disorder caused by pathogenic variants in the cystic fibrosis transmembrane conductance regulator (*CFTR*) gene and primarily affects the respiratory, digestive, and reproductive systems. Globally, CF is most prevalent among European ancestry, with an incidence rate of approximately 1/2500 to 1/3500. In China, the incidence is about 1/128,000. However, CF is not extremely rare in the Chinese population; rather, its prevalence is significantly underestimated. The *CFTR* mutation spectrum in China is highly unique, characterized by an extremely low frequency of p.Phe508del. Instead, region-specific mutations such as p.Gly970Asp, p.Ile1023Arg, and p.Arg553Ter predominate, alongside a high proportion of splicing variants and complex rearrangements. A significant proportion of Chinese CF patients primarily present with CF-like phenotypes within the CF-related disease spectrum (such as congenital bilateral absence of the vas deferens and pseudo-Bartter syndrome), exhibiting overlapping features with classic CF but lacking typical respiratory-dominant symptoms. This review examines how these atypical symptoms deviate from the diagnostic pathways established in Western countries. Establishing localised data and functional platforms is a prerequisite for achieving precision medicine. Achieving a transition from symptom-focused care to defect-correcting therapy will require coordinated multicenter collaboration and sustained infrastructure development.

## 1. Introduction

Cystic fibrosis (CF) is a systemic disease caused by mutations in the cystic fibrosis transmembrane conductance regulator (*CFTR*) gene, affecting multiple organs and tissues including the respiratory tract, pancreas, hepatobiliary system, gastrointestinal tract, reproductive organs, and the immune system. The central pathophysiology is impaired apical anion secretion (Cl^−^/HCO_3_^−^) and disrupted electrolyte homeostasis, which lead to airway surface liquid (ASL) dehydration and acidification, abnormal mucus architecture, impaired mucociliary clearance, chronic airway colonization, and neutrophil-predominant inflammation. These processes culminate in progressive structural lung damage. Over the past decade, the CF treatment paradigm has shifted from “symptomatic delay” to precision medicine targeting “correction of the fundamental defect”: CFTR correctors/potentiators have demonstrated significant improvements in lung function, acute exacerbations, and nutritional status in both clinical trials and real-world studies [1,2]. Concurrently, whole-genome sequencing of *CFTR* in large CF cohorts has revealed a more comprehensive variant landscape, directly informing variant-specific therapeutic selection and genetic counseling [3].

CF is an autosomal recessive disorder, with mutations typically transmitted through carriers who generally exhibit no symptoms. Consequently, the condition can persist within populations over extended periods, even when prevalence is low. The presence of carriers serves to maintain the mutation across different populations. Classic textbooks suggest CF primarily affects European ancestry populations, with an incidence rate of approximately 1 in 2500 to 1 in 3500 in North America and Europe, with a carrier frequency of about 1 in 25, while Asian populations exhibit significantly lower rates [4]. This “low incidence” has long been simplistically equated with “extreme rarity”, overlooking widespread underdiagnosis and misdiagnosis stemming from inadequate genetic testing, insufficient clinical awareness, and absent newborn screening. More crucially, differences in the *CFTR* mutation spectrum between ethnic groups reduce the sensitivity of traditional mutation profiling, further amplifying this phenomenon. Therefore, the prerequisite for cross-ethnic precision treatment lies in establishing cross-ethnic genetic infrastructure and functional validation systems, rather than simply extrapolating evidence from Western populations.

During the past decade, Chinese clinical case series and single-center and multicenter retrospective analyses have steadily accumulated [5,6,7,8,9,10,11]. This has been complemented by systematic studies of the genetic spectrum and functional research primarily involving Chinese CF patients [5,9,11,12,13,14,15,16,17,18,19,20]. Key findings in Chinese CF research during this period include: (1) systematic pedigree studies revealing high-frequency mutations such as p.Gly970Asp [5,9,11,12,13,14,15,16,17,18,19,20,21]; (2) identification of atypical phenotypes, including CF-like phenotypes (e.g., congenital absence of the vas deferens (CBAVD)) [6,8,9,10,11]; and (3) basic research expanding the functional boundaries of CFTR, such as the Wnt and nuclear factor kappa-B (NF-κB) pathways [22,23,24,25,26,27,28,29,30,31,32,33]; these advances have progressed from case summaries to multicentre cohort studies and translational research. These studies have not only characterized mutation patterns in Chinese CF patients (e.g., p.Gly970Asp and p.Arg553Ter represent previously characterised variants that are either highly frequent or clustered in China, whereas p.Ile1023Arg constitutes a founder mutation among Han Chinese in southern China) [9,11,14,16,21], but also revealed diverse organ system phenotypes and “CF-like” phenotypes [6,8,9,10,11]. Concurrently, a series of basic and translational studies have expanded the functional boundaries of CFTR at the pathway level, including β-catenin/Wnt, NF-κB, Hedgehog, vascular reactivity, and skeletal muscle aging [19,22,23,24,25,26,27,28,29,30,31,32,33]. NF-κB, a key transcriptional regulator in inflammation, drives neutrophilic inflammatory cascades and exacerbates tissue damage. Developmental and repair pathways, such as β-catenin/Wnt and Hedgehog, are essential for epithelial differentiation, polarity maintenance, and remodeling. These pathways significantly interact with fibrotic signals, such as TGF-β, which may contribute to the structural airway remodeling seen in the long-term progression of CF. These signaling pathways are closely associated with CF pathophysiology. Therefore, selective modulation of NF-κB and the rebalancing of the Wnt/Hedgehog-centered “repair-remodeling program” present promising therapeutic strategies beyond CFTR correction and enhancement [19,22,23]. We emphasise that, given existing CFTR modulators predominantly target Western common mutations such as p.Phe508del, a comprehensive understanding of mutations unique to the Chinese population (e.g., p.Ile1023Arg) and the functional impact of high-frequency mutations (e.g., p.Gly970Asp, p.Arg553Ter) is essential for precision therapy, as it directly influences CFTR modulator selection and gene therapy strategies [5,9,11,12,13,14,15,16,17,18,19,20,21].

## 2. CFTR: Structure, Function, and Mutation–Phenotype Correlations

### 2.1. Overview of CFTR Structure and Function

CFTR is a member of the ATP-binding cassette (ABC) transporter family and comprises two membrane-spanning domains (MSD1/2), two nucleotide-binding domains (NBD1/2), and a regulatory (R) domain enriched in phosphorylation sites [4,34]. R-domain phosphorylation lowers the NBD gating energy barrier, while ATP binding promotes NBD dimerization and drives channel opening; ATP hydrolysis and NBD dissociation are coupled to the closing process [35,36].

Its primary functions include the following: it acts as a Cl^−^/HCO_3_^−^ channel in the apical membrane of epithelial cells to regulate fluid and mucus viscosity in airway and gastrointestinal surfaces; it forms functional complexes with transporters like SLC26A9 to maintain epithelial ion homeostasis [31]; beyond ion transport, CFTR interacts with signaling proteins such as β-catenin and Dishevelled to modulate Wnt/β-catenin signaling and influence proliferation and differentiation [22,23,27]; and it participates in immune and inflammatory regulation by influencing monocyte adhesion, NF-κB activation, and Annexin A1 expression [26,30,32,37].

### 2.2. Classification of CFTR Mutations and Functional Defect Patterns

**Figure 1:**
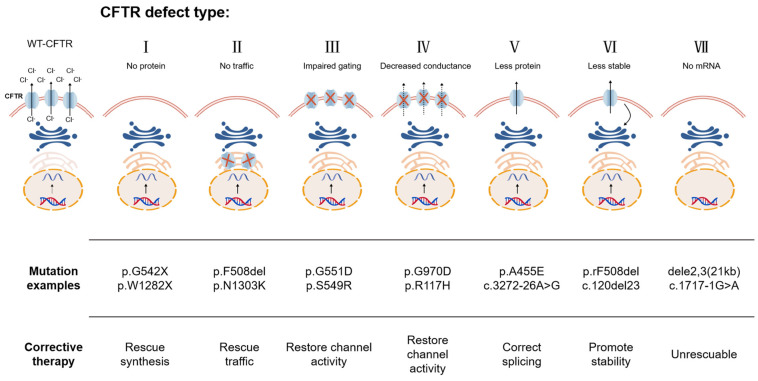
Classes of *CFTR* mutations [41]: Class I mutations prevent protein synthesis. Class II mutations result in misfolded proteins being degraded via the proteasome pathway. The red symbols in the figure indicate misfolded proteins retained due to failure to pass through the endoplasmic reticulum (ER) quality control mechanism, impairing CFTR protein transport. Class III mutations synthesize defective channel switches, with red symbols indicating impaired CFTR ion channels on the cell membrane. Class IV mutations manifest as reduced conductance, with dashed arrows indicating decreased ion transport efficiency. Class V mutations cause significant reduction in CFTR protein levels, with only minimal protein reaching the membrane. Class VI mutations increase CFTR endocytosis or decrease its recycling efficiency to the cell surface; downward curved arrows in the diagram indicate CFTR protein degradation upon reaching the cell membrane. Class VII mutations cause mRNA instability, which is not expected to be rescued by any modulator; created in BioGDP.

Based on protein processing and functional abnormalities, *CFTR* mutations are commonly categorized into Classes I–VI (with some literature adding Class VII, Figure 1) [4,34]: Class I: No protein production (e.g., nonsense mutation p.Arg553Ter) [38]; Class II: Abnormal folding and endoplasmic reticulum retention (prototypical example: p.Phe508del) [34]; Class III: Defective channel gating, resulting in synthesis of a defective channel protein; Class IV: Reduced ion permeability and impaired transport capacity; Class V: Reduced protein levels due to abnormal mRNA splicing, with only minimal protein reaching the membrane (e.g., splicing abnormalities associated with c.1210-3C>G and the 5T transmembrane-pre-polymer T tract) [39]; Type VI: Decreased membrane protein stability; Type VII: Difficulty in proper folding or severe structural disruption leading to mRNA instability (unresponsive to drug rescue) [40,41].

Studies from Chinese patients indicate that many “non-p.Phe508del” mutations exhibit complex functional profiles: for example, p.Gly970Asp demonstrates severe functional loss with abnormal protein processing in specific cell types [16,20,21]; complex rearrangements (mutations involving large-scale deletions/insertions that alter gene structure) and compound heterozygous mutations (where different mutations exist on both alleles) can trigger the superposition of multiple mechanisms (simultaneous action of several defective pathways) [17,18,19,20]. These points are particularly critical for assessing the target population for CFTR modulators and subsequent gene therapy strategies.

### 2.3. Correspondence Between Mutation, Protein Function, and Clinical Phenotype in the Chinese Population

Multiple Chinese cohort studies indicate that p.Gly970Asp is one of the most common or high-frequency mutations in Chinese CF patients, observed in both typical CF children and adult cases of CBAVD and spermatogenesis disorders [9,11,16,18,21]. It is a missense variant and classified as a Class IV mutation, consistent with an ion permeability defect. The affected residue lies near the C-terminus within the transmembrane–regulatory region of CFTR. Substitution of glycine with aspartic acid introduces a charged, bulkier side chain, which can alter local physicochemical properties and destabilize CFTR tertiary structure and channel conformational dynamics. These changes may impair endoplasmic reticulum folding and trafficking and/or reduce channel open probability, ultimately manifesting as a significant loss-of-function effect [16,20,21]. Consistent with in vitro predictions, structural modeling, and prior functional annotations of *CFTR* missense variants, analogous substitutions often reduce protein stability and compromise channel activity. Clinically, it correlates with a more severe respiratory phenotype, male infertility, and multi-organ involvement [9,11,16,18,21]. In certain Chinese/East Asian populations, p.Ile1023Arg has been proposed as a founder mutation associated with classic CF phenotype and severe pulmonary disease. Located within the MSD2 region, this mutation may simultaneously impact channel gating and protein stability [14]. Splicing-related mutations (e.g., c.1210-3C>G/5T) account for a proportion of Chinese CF cases. Functional splicing analysis indicates that c.1210-3C>G, when co-occurring with the 5T poly-T tract, causes significant abnormalities in CFTR mRNA splicing and reduced protein expression. Clinically, this may manifest as atypical CF or CF-like phenotypes (e.g., mild respiratory symptoms or CBAVD) [8,15,18,39]. Case reports indicate that small deletions and complex rearrangements (e.g., c.753_754delAG, compound large-segment deletions) often result in severe protein truncation or structural disruption, presenting as typical CF phenotypes accompanied by severe pulmonary and nutritional issues in infancy or childhood [10,17,29,38,42]. The aforementioned genotype–phenotype associations are based on clinical observation data from Chinese patients [6,9,11,16,21], supplemented by functional experimental results for certain mutations [20,21].

Overall, the Chinese *CFTR* variant spectrum is characterized by predominance of non-p.Phe508del variants, with enrichment of p.Gly970Asp and a substantial contribution from splicing variants and complex rearrangements. Accordingly, building a mutation–phenotype database and functional stratification framework for China requires approaches that do not simply replicate the p.Phe508del-centered Western model [5,6,7,8,9,11,12,13,14,15,16,18,20].

## 3. CF and Ethnicity: Prevalence, Mutation Spectrum, and Clinical Features

### 3.1. European Ancestry Populations: Classic CF Pattern Centered on p.Phe508del

In North America and Europe, the prevalence of CF among European ancestry is approximately 1 in 2500 to 1 in 3500, with a carrier frequency of about 1 in 25. The mutation spectrum is absolutely dominated by p.Phe508del, with the p.Phe508del allele frequency exceeding 60–70% in some countries [4]. Clinical features manifest in childhood with typical symptoms of thickened mucus, recurrent bronchopulmonary infections, and pancreatic exocrine insufficiency; many patients develop severe bronchiectasis or respiratory failure by young adulthood, and a lung transplant may be required as a treatment option. CFTR modulators (particularly the triple combination of Elexacaftor/Tezacaftor/Ivacaftor) significantly improve lung function, nutritional status, and survival prognosis in the p.Phe508del-related population [43,44]. Mechanistically, p.Phe508del destabilizes NBD1 folding and disrupts interdomain assembly, enhancing ER quality control and ER-associated degradation, impairing maturation glycosylation, and markedly reducing trafficking to the membrane. Even when some protein reaches the surface, gating and stability remain abnormal [35,36]. This multifactorial defect underlies the limited efficacy of single agents and supports the rationale for combination therapy. The “classic” European-ancestry phenotype has shaped prevailing diagnostic criteria and management pathways, but this model does not directly generalize to Chinese populations [4,45,46].

### 3.2. African Populations: Severe Phenotypes and Diagnostic Delays

CF prevalence in African populations is lower than in European ancestry populations but significantly higher than current estimates for Asians [4,46]. Available evidence suggests that the African CF population experiences diagnostic delays, significant phenotypic heterogeneity, and a wide range of mutations. Clinically, CF in Africa is primarily characterized by chronic suppurative airway disease (recurrent pneumonia/bronchiectasis, chronic rhinosinusitis, bacterial colonization), often accompanied by multisystem involvement, such as pancreatic exocrine insufficiency, malnutrition, and growth retardation [46,47]. CF in Africa has historically been hindered by a structural issue of “low recognition—low diagnosis—low registration” [47,48]. South African registry studies reveal a younger patient population with greater ethnic diversity. Factors such as malnutrition and the presence of drug-resistant bacteria (e.g., MRSA) correlate with more severe pulmonary disease, suggesting that basic nutrition and infection management may have a more significant impact on disease progression in resource-limited settings [48]. At the genetic level, a North African review identified significant regional and ethnic variation in *CFTR* pathogenic variants. In addition to the common p.Phe508del mutation, several truncating and splicing site mutations exhibit clustered distributions across countries. High rates of consanguinity in the region further increase the burden of recessive pathogenic alleles, which may contribute to early-onset disease and multisystem involvement [47]. The prevalence of CF among African populations is estimated to be approximately 1 in 17,000, with diverse mutation lineages, including founder mutations such as c.3120 + 1G>A, which may account for up to 12% of alleles [49]. The mutation spectrum is more diverse, with markedly lower p.Phe508del frequencies than in Europe and higher proportions of regional founder mutations and truncating mutations [46]. Some cases present with earlier-onset severe pulmonary phenotypes, malnutrition, and higher early mortality rates. Inadequate newborn screening coverage and uneven distribution of healthcare resources lead to significant diagnostic delays. The proportion of patients receiving CFTR modulators is substantially lower than in American and European ancestry populations [45,46]. These data suggest that “low reported incidence” in any population does not equate to “true low prevalence,” but may reflect disparities in diagnostic capacity and healthcare systems.

### 3.3. Asia–Pacific Region: Represented by Japan, South Korea, Southeast Asia, and Chinese Populations

CF reporting in the Asia–Pacific region—including Japan, South Korea, Singapore, and parts of Southeast Asia—has increased in recent years [4,9,45]. Although case counts remain lower than in Europe and North America, they continue to rise with expanded newborn screening and increased use of whole-exome sequencing (WES). p.Phe508del is rare in these populations; instead, region-specific variants and missense/splice variants predominate [5,7,8,9,11,12,14,15,16,18,19,20]. Phenotypically, classic CF coexists with “CF-like” phenotypes, the latter including single-system involvement (e.g., isolated CBAVD, recurrent sinusitis, CF-related liver disease) [6,8,9,10,11,15,29,38].

### 3.4. Characteristics of Chinese CF Patients in the Global Landscape

Although case counts remain lower than in Europe and North America, they continue to rise with expanded newborn screening and increased use of WES. p.Phe508del is rare in these populations; instead, region-specific variants and missense/splice variants predominate [5]. Epidemiological modeling estimates a CF incidence of ~1/128,000 in China—lower than in European-ancestry populations but not exceptionally rare [7]. Combining pediatric and adult cohort data from China reveals that most affected children present during childhood with recurrent cough, wheezing, bronchiectasis, or severe pneumonia, often misdiagnosed as refractory asthma or bronchiectasis [5,6,8,9,10,11]. Gastrointestinal and nutritional manifestations (e.g., sticky stools, steatorrhea, and growth failure) are common in neonates and infants but are frequently attributed to nonspecific “chronic diarrhea” or “malnutrition”, delaying CF evaluation [5,6,9]. To date, a significant proportion of adolescent and adult “CF-like” cases exist, presenting with recurrent pancreatitis, pseudo-Bartter syndrome-like hypokalemic alkalosis, CF-associated liver disease, or isolated CBAVD [8,9,10,11,12,15,18,38,50]. CFTR is not merely a chloride channel, but a central “hub protein” within several critical signaling networks, including Wnt, NF-κB, autophagy, vasoregulation, and tumorigenesis [22,23,24,25,26,27,28,29,30,31,32,33]. Its interactome and localization platforms not only regulate chloride and bicarbonate transport but also interact with autophagy–lysosomal systems, inflammatory transcription pathways (e.g., NF-κB), and repair-remodeling pathways (e.g., Wnt/Hedgehog) via PDZ-associated complexes. As a result, CFTR dysfunction leads to widespread systemic effects [19,22,23,24,25,26,27,28,29,30,31,32,33]. In sweat glands, it causes salt loss, which, under certain conditions, can progress to pseudo-Bartter syndrome. In the hepatobiliary system, it results in secretory abnormalities exacerbated by inflammation and fibrosis. In skeletal muscle, CFTR dysfunction disrupts calcium homeostasis and inflammatory gene regulation, which, in conjunction with systemic inflammation and impaired autophagy, contributes to muscle aging phenotypes. In epithelial tissues, such as the gastrointestinal tract, persistent inflammation and repair imbalances, along with altered proliferative signaling, may increase susceptibility to tumors [22,23,24,25,26,27,28,29,30,31,32,33]. Given the high heterogeneity of *CFTR* variants in Chinese CF patients, along with their distinct profiles compared to Western populations, the activation and compensatory responses within these pathways may vary more significantly, contributing to a broader phenotypic spectrum.

Using gnomAD v4.1.0, allele frequencies of key *CFTR* variants differ markedly between East Asian and global populations. In East Asians, p.Gly970Asp has an allele frequency of 0.0001581 (7/44,284), substantially higher than the global frequency of 0.0000059 (9/1,515,888). In contrast, p.Arg553Ter is less frequent in East Asians (0.0000223; 1/44,766) than globally (0.0000971; 156/1,606,458), with the highest frequency observed in Europeans (0.0001176; 138/1,173,372). Similarly, p.Phe508del is rare in East Asians (0.00002232; 1/44,802) compared with the global frequency (0.01193; 19,237/1,612,320) and the European frequency (0.0143048; 17,773/1,242,448). Notably, p.Ile1023Arg, as well as many splice-site variants and complex rearrangements, is not yet represented in this database.

The “classical” CF phenotype described in European ancestry populations—driven by high-frequency variants such as p.Phe508del and a characteristic pulmonary–pancreatic presentation—has historically dominated cohort studies, newborn screening panels, and enrolment in therapeutic trials. This evidence base has shaped current diagnostic criteria and clinical heuristics. In practice, clinicians may apply pattern-based “prototype matching” when deciding whether to pursue sweat chloride testing, extended *CFTR* sequencing, or functional assessment.

In Chinese patients, the *CFTR* variant spectrum is more dispersed and includes many variants that are rare in European ancestry populations. Coupled with limited access to sweat testing and lower clinical familiarity with CF, this increases the likelihood that non-classical presentations are missed at initial points of care. Patients presenting with recurrent wheeze or cough may be managed within the asthma spectrum, whereas those with chronic diarrhoea or malnutrition may be identified as having more common gastrointestinal disorders. These factors contribute to systematic underdiagnosis and misdiagnosis [4,5,6,8,9,10,11,45,46]. Collectively, Chinese CF is characterized by a lower reported incidence, a highly distinct variant spectrum, and broad phenotypic diversity, supporting the need for diagnostic and management pathways tailored to China rather than direct adoption of p.Phe508del-centered Western frameworks.

## 4. Clinical Phenotypes and Mutation Spectrum of Chinese Patients with Cystic Fibrosis

### 4.1. Epidemiology and Undiagnosed Cases

A systematic estimation based on population variant frequencies and Bayesian modeling suggests that, given China’s population size, the expected number of individuals with CF is far higher than current clinical reporting implies [7]. Combining multiple case summaries and single-center cohort analyses [6,8,9,10,11], several key issues emerge: (1) Diagnostic delay: Many children undergo *CFTR* gene testing only after years of recurrent pulmonary infections or bronchiectasis [6,8,9,11]; (2) Incomplete neonatal screening coverage: Most regions in China have not yet incorporated CF into routine newborn screening, making it difficult to identify mild or atypical CF cases in Chinese birth cohorts; (3) Regional disparities: Reported cases are predominantly concentrated in large pediatric centers or a few developed coastal cities, with patients in western and grassroots areas being more prone to underdiagnosis or misdiagnosis. These factors collectively lead to a systematic underestimation of CF’s true prevalence in China, with little prospect of rapid improvement [7,9]. Diagnosis of CF in Chinese paediatric patients is frequently delayed. Studies report a median interval of 10 years from symptom onset to diagnosis among Chinese children [6]. In Chinese paediatric cohorts, the mean delay from initial presentation to diagnosis is approximately 5.7 years [9]. Earlier single-centre series reported even longer delays, averaging ~11 years when calculated case-by-case from age at symptom onset to age at diagnosis [8]. Common misdiagnoses in Chinese paediatric practice include attributing CF-related recurrent wheezing to asthma and interpreting diarrhoea and malnutrition due to pancreatic exocrine insufficiency as chronic indigestion [6,8]. Beyond sweat chloride testing and genotyping, functional assays (e.g., nasal epithelial or rectal organoid electrophysiology) can clarify pathogenicity of rare variants and predict modulator responsiveness—capabilities that are particularly critical for a population with a highly dispersed variant spectrum. Strategically, a “genome sequencing–phenotype–therapy selection” pathway remains applicable in China; positioning sequencing as an early diagnostic step, rather than a late add-on, may reduce delays associated with mutation-panel negativity [3].

### 4.2. Pediatric Cohorts: Respiratory, Gastrointestinal, and Nutritional Phenotypes

Cohort studies from Beijing, Shanghai, and other centers have characterized pediatric CF phenotypes in China. Most children present with respiratory manifestations, including chronic cough, wheeze, recurrent lower respiratory tract infections, and imaging findings such as bronchiectasis, atelectasis, or diffuse tree-in-bud opacities; in some patients, chronic colonization with Gram-negative bacteria (e.g., Pseudomonas aeruginosa) is observed [5,6,8,9,11]. Gastrointestinal and nutritional abnormalities are also common, including steatorrhea, chronic diarrhea, growth failure, hypoalbuminemia, and deficiencies of fat-soluble vitamins [5,6,9,10]. Case reports describe electrolyte and acid–base abnormalities resembling pseudo-Bartter syndrome—hypokalemia and hypochloremic metabolic alkalosis—in CF infants [10,50]. Other complications include hepatobiliary disease, cirrhosis, and even acute liver failure, suggesting CF-related liver disease is also not uncommon among Chinese pediatric patients [38].

Phenotype severity often aligns with variant type. Patients carrying p.Gly970Asp or compound splicing/truncating variants frequently show more typical and severe disease [6,9,11,16,21], whereas milder missense variants or partial splicing defects are more often associated with atypical disease or single-system involvement [8,9,15,18,39].

### 4.3. Male Reproductive Phenotype and CBAVD

Multiple Chinese cohort studies have specifically examined the patient group with “CBAVD + *CFTR* mutation” [12,14,15,18]. Beyond p.Gly970Asp, multiple splicing variants, missense mutations, and multiple heterozygous variants cluster in males with CBAVD [12,14,15,18]; male *CFTR*-related disorders form a clinical continuum. The mildest end comprises isolated CBAVD, which clinically presents only as obstructive azoospermia/oligozoospermia accompanied by features of accessory gland involvement, including low semen volume, acidic pH, and fructose deficiency, without respiratory or gastrointestinal symptoms. In Chinese CBAVD cohorts, *CFTR* variants are relatively common, and a considerable proportion of individuals carry CF-pathogenic variants (cohort plus meta-analysis suggesting that approximately 14.8% carry ≥1 CF-pathogenic variant), indicating that some cases are not isolated reproductive tract defects but rather mild phenotypes of *CFTR*-related disorders [3,17]. The intermediate segment of the spectrum manifests as a CF-like phenotype with CBAVD and mild/intermittent multisystem involvement, which may include chronic rhino-rhinosinusitis, recurrent lower respiratory tract infections or bronchiectasis, and elevated sweat chloride or a predisposition to electrolyte disturbances [7]. The severe end shows multisystem manifestations resembling classic CF, characterised predominantly by bronchiectasis and persistent airway infection/colonisation, and may be accompanied by malabsorption, pancreatic exocrine insufficiency, or other glandular secretory disorders, resulting in a male clinical spectrum dominated by respiratory involvement, with digestive involvement relatively uncommon but markedly heterogeneous [6,33]. Notably, p.Gly970Asp is frequent in pediatric CF and has also been associated with spermatogenic disorders in some infertility cohorts [21]. These findings indicate a substantial reproductive-dominant spectrum of *CFTR*-associated disease in China that may be missed by diagnostic criteria optimized for classic pulmonary presentations.

### 4.4. Other Organ Complications

A Chinese child with a homozygous p.Arg553Ter nonsense mutation presented primarily with progressive liver failure and portal hypertension, accompanied by pancreatic insufficiency, indicating that severe hepatic phenotypes in CF also occur in China [38]. Some infants or children present with hypokalemia, hypochloremic alkalosis, and dehydration, exhibiting pseudo-Bartter syndrome-like manifestations that are subsequently confirmed by genetic testing to harbor *CFTR* pathogenic variants [10,50]. Pseudo-Bartter syndrome is fundamentally characterised by recurrent hyponatraemia, hypochloraemia, and hypokalaemia with metabolic alkalosis resulting from impaired salt reabsorption in sweat glands. This disturbance is often exacerbated under stressors such as fever, hot weather with profuse sweating, inadequate intake, or infection. Clinically, it is easily mistaken for primary Bartter syndrome, thereby delaying recognition of CF. One report described three infants admitted with “Bartter-like hypokalaemic alkalosis” who were diagnosed with CF through exome sequencing combined with sweat chloride testing; two were compound heterozygotes and one carried a homozygous missense variant. The study also identified two potentially pathogenic novel variants (c.1526G>C and c.3062C>T), suggesting that pseudo-Bartter syndrome can serve as an early clue to CF in the Chinese population and that genetic confirmation is often required [10]. CF-related pseudo-Bartter syndrome most commonly has an onset at 1–36 months of age, whereas the age at diagnosis may be delayed to 3–144 months. Most affected children exhibit additional systemic manifestations, frequently including recurrent respiratory tract infections or persistent pneumonia, malnutrition, and growth and developmental delay; some develop pancreatitis or suspected pancreatic exocrine insufficiency. At the genetic level, p.Gly970Asp is the most frequently reported variant [50]. Therefore, in infants with recurrent hyponatraemia/hypokalaemia/hypochloraemia accompanied by metabolic alkalosis—particularly those with growth impairment or respiratory infections—CF should be included in the differential diagnosis, and *CFTR* genetic testing (exome sequencing when necessary) together with sweat chloride and/or CFTR functional assessment should be performed as early as possible to reduce misdiagnosis and diagnostic delay. These findings underscore that CF in the Chinese population is not merely a “pulmonary disease” but a classic multisystem disorder, particularly requiring systematic follow-up and intervention for liver and electrolyte balance [10,24,26,38].

### 4.5. China-Specific or High-Frequency Mutation Spectrum

In multiple CF pediatric and adult CBAVD cohorts, p.Gly970Asp has been reported as the most common or high-frequency mutation [9,11,16,18,21], considered one of the “hallmark” variants of Chinese CF. Functional studies indicate it is a distinct loss-of-function mutation, potentially associated with protein folding and transport abnormalities [20,21]. Additional typical mutations include p.Ile1023Arg, a founder mutation in southern Han Chinese populations. The mutant protein exhibits transport defects, suggesting dysfunction arises from reduced expression of fully glycosylated protein on the cell surface [14]. p.Arg553Ter and other nonsense mutations correlate with the classic severe CF phenotype, including liver failure and severe malnutrition [38]. Splicing-related variants (e.g., c.1210-3C>G + 5T) cause abnormal *CFTR* mRNA splicing, placing a significant proportion of patients on a continuum “between CF and CF-like” [8,9,15,18,23]. Complex deletions and compound heterozygosity (including de novo large-segment deletions and small deletions, e.g., c.753_754delAG) typically involve significant protein structural disruption and result in more severe clinical phenotypes [10,17,19,29,38,42]. A WES study of two consanguineous families primarily identified homozygous point mutations (e.g., p.Arg334Trp and the novel variant p.Val470Glu). Each family carried a homozygous *CFTR* variant: Family 1 harboured the previously reported pathogenic variant p.Arg334Trp, whereas Family 2 carried the newly identified p.Val470Glu, located near a canonical polymorphic site and described as a “polymorphism-associated locus” [19]. Sanger validation confirmed homozygosity in three affected children, with both parents being heterozygous carriers, consistent with autosomal recessive segregation in the context of consanguinity [19]. The *CFTR* variant spectrum in Chinese patients with CF is dominated by single-nucleotide variants and small insertions/deletions, whereas copy-number variants (CNVs) have been reported at a relatively low frequency in the existing literature. The most representative evidence to date comes from a study of a Chinese child with CF: after conventional exome sequencing failed to explain the phenotype, multiplex ligation-dependent probe amplification (MLPA) and breakpoint sequencing identified a homozygous complex rearrangement/large deletion involving *CFTR* exon 20 (c.3140-454_c.3367 + 249del931ins13; p.R1048_G1123del). Based on this finding, the authors emphasised that MLPA should be routinely incorporated into CF pathogenic-variant screening in the Chinese population to capture CNVs [17]. This “single-case discovery” suggests that CNVs will be systematically underestimated if analyses rely solely on targeted or exome sequencing without a robust CNV-calling workflow. In another analysis of a Chinese CBAVD cohort, the authors explicitly recommended “whole-exome and flanking-region sequencing with detection of large rearrangements and deep intronic pathogenic variants”; however, their reported data were largely organised around single-nucleotide variants and small insertions/deletions, indirectly reflecting the lack of systematic statistics on CNV frequency [18]. Therefore, regarding “occurrence frequency,” the available evidence supports the existence of CNVs in Chinese CF patients, but reports remain sporadic and the true frequency is likely substantially underestimated. Future multicentre cohorts adopting MLPA uniformly or implementing NGS pipelines with reliable CNV detection will be required to generate comparable CNV detection rates and allele frequencies.

Compared to the “p.Phe508del-dominant” pattern in European ancestry, the molecular basis of CF in China resembles a multi-peak distribution characterized by multiple “moderate-to-high-frequency region-specific variants + numerous rare variants”; the multimodal distribution of the Chinese *CFTR* variant spectrum may therefore stem from the absence of a single dominant mutation. In other words, different regions harbour their own variants with intermediate-to-high frequencies, while substantial genetic diversity allows numerous low-frequency variants to coexist, collectively producing a relatively dispersed variant-frequency landscape [5,8,9,11,12,13,14,15,16,18,19,20]. This distribution has major implications for modulator eligibility assessment and the design of gene-based therapies.

## 5. Current Status of CF Treatment and Translational Research Directions

### 5.1. Traditional Treatment and Comprehensive Management

Before CFTR modulators (often benchmarked by the 2012 approval of ivacaftor), CF care in Europe and the United States relied on comprehensive management aimed at suppressing infection, controlling inflammation, enhancing mucus clearance, and preventing or treating acute exacerbations. Standard care includes daily airway clearance therapies (e.g., chest physiotherapy and oscillatory positive expiratory pressure) and inhaled mucolytic or mucus-modifying agents (notably hypertonic saline and dornase alfa) to reduce sputum viscosity, improve ventilation, and lower exacerbation risk. For chronic airway infection—particularly Pseudomonas aeruginosa—long-term inhaled antibiotics (e.g., tobramycin, amikacin, polymyxins) are commonly used for suppressive therapy. These approaches are paired with standardized microbiological surveillance (sputum and/or throat cultures) and iterative regimen adjustment. Overall, this strategy aims to slow lung function decline while balancing toxicity and the high treatment burden imposed by progressive bronchiectasis [51,52,53,54].

Parallel to the above pulmonary therapies lies the ongoing correction of the systemic phenotype “malabsorption–malnutrition–complication spectrum”: European and American guidelines and practices emphasize intensive nutritional management (individualized energy intake, fat-soluble vitamin supplementation) and pancreatic enzyme replacement therapy for exocrine pancreatic insufficiency to maintain body composition and growth development, forming a positive coupling with pulmonary outcomes. Simultaneously, structured screening and stratified interventions target CF-related complications such as diabetes, liver disease, bone disorders, and intestinal obstruction. Care is typically delivered by multidisciplinary teams (respiratory, nutrition, infectious disease, endocrinology, psychology, and social support) within specialized centers. For acute exacerbations, antibiotics remain the cornerstone (oral or intravenous regimens selected based on historical pathogens and clinical response), whereas adjunctive systemic corticosteroids are used more cautiously and selectively [52,55,56,57,58].

In China and across the Asia–Pacific region, most patients still rely on conventional comprehensive therapy, including airway clearance, hypertonic saline nebulization, antibiotic management, nutritional support, pancreatic enzymes, fat-soluble vitamin supplementation, and supportive care for complications such as liver disease and electrolyte disturbances [4,5,6,8,9,10,11]. Lung and liver transplantation have been performed in select severe cases, but overall access remains limited.

### 5.2. CFTR Modulators: From p.Phe508del to Multi-Mutation Indications

The high frequency of p.Phe508del in European and American CF populations has facilitated more robust drug development and clinical evidence accumulation centered on this mutation. Large-scale randomized controlled trials abroad have demonstrated that CFTR modulators targeting p.Phe508del and specific gating/residual function mutations (e.g., Ivacaftor, Lumacaftor/Ivacaftor, Tezacaftor/Ivacaftor, and Elexacaftor/Tezacaftor/Ivacaftor(ETI)) significantly improve lung function, nutritional status, and quality of life while reducing acute exacerbation rates [40,43,44,46]. ETI demonstrates “mutation-correcting” efficacy in patients with one or two p.Phe508del mutations; some models project it could extend median lifespan beyond 50 years [40,43,44]. It is important to note that “high-efficiency modulators” do not imply complete disease reversal: pre-existing structural bronchiectasis and airway remodeling may limit reversibility. Additionally, residual inflammatory and infectious burdens, shifts in metabolic complication profiles, and long-term safety (e.g., liver function, psychiatric/drug interactions) require ongoing registry data to be fully characterized [2].

However, the vast majority of these trials were conducted in American and European ancestry populations, and indications are highly dependent on p.Phe508del and a few specific mutations. This makes it difficult to directly apply these findings to the Chinese patient population, which predominantly carries p.Gly970Asp and multiple splicing/complex mutations [5,7,9,11,12,14,15,16,18,20]. The key reason ETI is “difficult to apply effectively” is not because it is theoretically unusable, but rather because of the combined constraints of evidence extrapolation, genotype background, and health-system accessibility. First, although p.Gly970Asp has been included on the FDA-recognised list of ETI “responsive variants,” such indication expansions rely largely on in vitro validation rather than adequately powered clinical trials specifically targeting this variant. The FDA has explicitly allowed in vitro evidence to support designation of “responsive” variants, and large-scale in vitro screening indicates substantial heterogeneity in the magnitude of CFTR functional rescue across rare variants, with variable therapeutic effects [59]. Second, many Chinese patients are compound heterozygotes; if, in addition to p.Gly970Asp, they carry a class I variant or other low-responsiveness alleles, the total amount of recoverable CFTR protein may be markedly constrained, leading to weaker clinical benefit than expected under evidence paradigms largely built around p.Phe508del [5,7,9,11,12,14,15,16,18,20]. Third, longstanding diagnostic delays and limited availability of key tests (e.g., sweat chloride) in China mean that ETI may be initiated when structural lung damage is more advanced and the reversible component is smaller. Finally, in countries or regions without formal reimbursement and stable supply, CFTR modulators often depend on out-of-pocket purchase, donations, or informal channels, resulting in low coverage and discontinuous follow-up and monitoring, which further undermines real-world effectiveness and safety management [60]. Taken together, under a high-prevalence p.Gly970Asp background, the principal bottleneck for ETI reflects a scenario of being “potentially responsive but difficult to implement at scale in a standardised manner and to achieve stable clinical benefit”. Based on existing literature, China currently lacks systematic studies evaluating the efficacy of CFTR modulators in locally prevalent *CFTR* mutations, particularly p.Gly970Asp, p.Ile1023Arg, and complex splicing mutations. Current efforts primarily involve foundational experiments and early-stage in vitro drug screening, laying the groundwork for future ex vivo “theratyping” and personalized medication [13,20,21,61,62].

Advances in cryo-electron microscopy and computational chemistry are accelerating structure-guided modulator discovery. One cell study combined ultra-large-scale docking (~155 million molecules) with electrophysiology, structural validation, and medicinal chemistry optimization to identify potent potentiators binding to the potentiator pocket. The same pocket was also found to accommodate inhibitors, highlighting the feasibility of co-site pharmacology and offering strategies to improve ivacaftor-like compounds’ pharmacokinetics and tissue distribution [63].

Even in the modulator era, airway clearance, inhaled therapies, infection control, and nutritional management remain key determinants of stability and long-term outcomes for many patients. A critical clinical shift is the integration of conventional therapies with genotype-directed treatments while accounting for inflammatory state and microbiologic dynamics. The interaction between inflammation and modulator response is particularly complex. For example, TNF-α plus IL-17 has been reported to enhance modulator-induced anion secretion by increasing intracellular Cl^−^/HCO_3_^−^ reserves via p38 MAPK signaling, whereas common anti-inflammatory drugs attenuate this effect [64]. This finding does not advocate “preserving inflammation,” but rather suggests shifting anti-inflammatory strategies from “suppressing all inflammation” to “preserving necessary ion secretion drivers without exacerbating structural damage.” Such approaches should be guided by quantifiable endpoints (e.g., ASL viscosity/hydration, ion flux, biomarkers).

A 2025 study elucidated the pore-blocking mechanism of (R)-BPO-27 with structural clarity, providing actionable structural coordinates for selective inhibition. For the CF field, such research is significant because it makes the “structure–function–efficacy” loop truly computable and iterable, establishing a methodological foundation for next-generation modulator combinations (including individualized chemical optimization) [65].

### 5.3. Novel Small-Molecule CFTR Modulators Derived from Traditional Chinese Medicine

Compound screening based on traditional Chinese medicine identified natural small molecules with potential to directly modulate CFTR function. Oridonin, a small molecule isolated from the Chinese herb Hedyotis diffusa, was confirmed as a CFTR inhibitor capable of altering CFTR channel activity [61]. Separately, an activity profiling study of anti-diarrheal Chinese medicine (Rhodiola kirilowii Maxim) identified two natural small molecules, EGCG and ECG, that inhibit CFTR-mediated chloride currents [62]. These studies provide candidates for the development of more affordable CFTR modulators, potentially better suited for specific populations (such as non-CF patients with diarrhea induced by *CFTR*-related microbiota), though the translation to CF patients remains at a very early stage [61,62].

### 5.4. Gene Therapy and Nucleic Acid Therapeutics: From Concept to Early Clinical Trials

**Figure 2:**
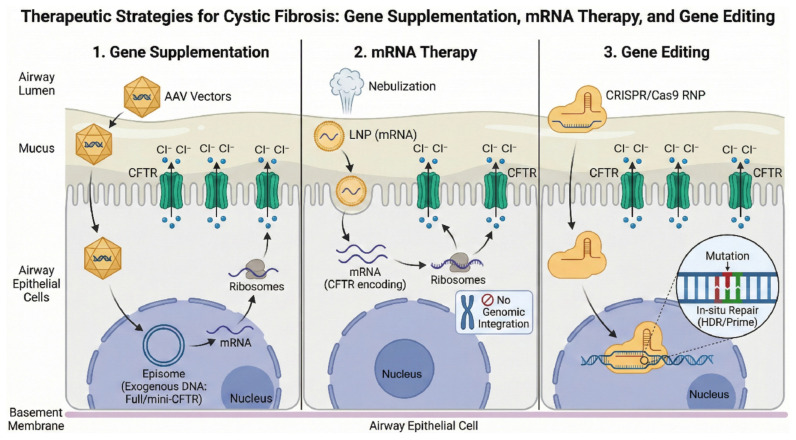
Schematic diagram of gene therapy.

In the CF field, gene therapy primarily encompasses the following strategies (Figure 2): (1) Gene replacement therapy: Delivery of the full-length or “mini-*CFTR*” gene via viral vectors (e.g., adeno-associated virus, AAV) or non-viral vectors to achieve sustained expression [45]; (2) mRNA therapy: Delivery of mRNA encoding normal *CFTR* via nebulized inhalation to achieve genotype-independent protein expression [46,66]; (3) Gene editing: Utilizing technologies like CRISPR/Cas9 to repair pathogenic *CFTR* mutations in situ, currently primarily confined to in vitro and animal studies [45,46].

Inhaled mRNA represents one of the closest-to-clinical, genotype-independent strategies, with its primary advantage being theoretical independence from the patient’s *CFTR* genotype, thereby covering the population that cannot be treated with modulators. A recent Phase 1/2 randomized double-blind clinical trial demonstrated that the nebulized mRNA drug MRT5005 was generally well tolerated in adult CF patients. Although no significant improvement in FEV_1_ was observed, it validated the feasibility of “lung mRNA delivery” [66]. Although there are multiple pathways for CF gene therapy (gene supplementation, mRNA, gene editing), the bottlenecks are equally clear, focusing on effective delivery, sustained expression/repair, and immune safety. First, airway delivery efficiency is restricted by multiple barriers: the high viscosity of CF airway mucus, purulent secretions, and biofilms significantly reduce vector diffusion; chronic inflammation leads to epithelial remodeling and increased cellular heterogeneity, reducing the exposure and transduction probability of target cells (basal cells/secretory cells). Additionally, CFTR must reach a certain “functional threshold” in the airways to yield clinical benefits; insufficient delivery may result in sub-threshold expression, leading to unstable efficacy, and the reversibility window varies across different stages of structural lung damage [66]. Second, persistence of expression and cell turnover are core challenges: airway epithelial renewal and tissue repair cause non-integrating vectors or free-expressed transgenes to gradually be lost; if permanent repair through integration or gene editing is pursued, it is essential to efficiently cover long-lived progenitor cells and avoid the risk of insertional mutations. For AAV, there are also packaging capacity limitations (difficulty in accommodating the full *CFTR* and regulatory elements). Third, immune responses significantly affect safety and re-administration: previous infections and natural exposure can lead to anti-AAV neutralizing antibodies, limiting re-dosing; local or systemic immune activation can cause worsened inflammation, clearance of transduced cells, and toxicity risks. While mRNA therapy offers advantages independent of genotype, mRNA and lipid nanoparticles may trigger innate immune recognition, and protein expression is transient, requiring repeated dosing and facing long-term tolerance and adherence issues [66]. Gene editing also adds risks such as off-target effects, large deletions/chromosomal rearrangements, DNA damage response, and low efficiency in delivering donor templates and achieving homologous repair; in an inflammatory microenvironment, these risks and efficiency issues may be further amplified. Overall, CF gene therapy’s transition from “concept feasibility” to “clinically repeatable and scalable” hinges on overcoming airway barriers, targeting cells effectively, improving progenitor cell coverage, and achieving immune-controllable long-term dosing and safety monitoring. Therefore, future breakthroughs are more likely to emerge from ‘combination strategies’: for example, first enhancing delivery efficiency by modifying the mucus/inflammatory microenvironment and then using mRNA to achieve greater functional restoration, or combining with alternative pathways (e.g., SLC26A9) to increase systemic redundancy for ASL hydration [66,67]. The latest review also systematically summarizes the progress and challenges in CF gene therapy regarding vectors, delivery routes, and safety [45].

For China, gene-based therapies may be particularly relevant because a substantial subset of patients carry variants that may not respond to existing modulators (e.g., nonsense variants and complex large deletions) or face access barriers due to cost and policy constraints. Patients with severe pulmonary disease or multisystem involvement may also benefit disproportionately from durable, mutation-agnostic therapies if delivery and persistence challenges can be addressed.

### 5.5. Challenges in CF Treatment for the Chinese Population

Several critical challenges currently exist in CF treatment in China: (1) Mismatched mutation profiles: Existing CFTR modulators primarily target p.Phe508del and a limited number of gating mutations, whereas the Chinese population predominantly carries p.Gly970Asp, p.Ile1023Arg, and splicing/complex variants [7,9,11,14,15,16,18,20,21]; (2) Lack of functional data: Systematic in vitro functional testing and drug response data are scarce for many mutation-specific or high-frequency mutations in the Chinese population, complicating their inclusion in “mutation-specific dosing” regulatory frameworks [13,18,19,20,21]; (3) Drug accessibility and cost: Even if triple-drug regimens like ETI become available in China, their pricing and medical insurance reimbursement policies will remain core factors affecting clinical accessibility; (4) Insufficient gene therapy infrastructure: This includes carrier production, standardized clinical trial platforms, and the establishment of long-term follow-up systems. Currently, the annual cost of ETI therapy abroad can reach hundreds of thousands of US dollars. Without adequate insurance coverage, the vast majority of Chinese patients would be unable to afford it; such economic factors will substantially constrain the clinical accessibility of new drugs in China. As of 2024, more than 90 medicines for rare-disease treatment have been included in the National Reimbursement Drug List (NRDL). Over the past decade, China’s rare-disease policy framework has evolved into a closed-loop pathway characterized by “catalog definition—guideline standardization—collaborative networks—research initiatives—accelerated review and approval—NRDL price negotiation and reimbursement entry—implementation via the dual-channel mechanism.” The catalog and clinical guidelines provide an institutional foundation for disease recognition and standardized diagnosis and treatment; collaborative networks and research programs strengthen early diagnosis and treatment and facilitate evidence generation; regulatory prioritization and expedited review improve access at the time of market entry; and, on the payer side, NRDL negotiation and the dual-channel mechanism enhance affordability and availability of rare-disease medications through coordinated payment and supply mechanisms. Therefore, advancing precision and gene therapy for CF in China is not solely a scientific challenge but also requires coordinated efforts in health policy and industrial development.

## 6. Limitations and Key Issues for Future Research

### 6.1. Common Limitations in Existing Data

Most Chinese studies remain small and are often single-center case series or case reports, limiting statistical power [5,6,8,9,10,11,12,17,19,29,38,42,50]; selection bias is substantial because cohorts are concentrated in tertiary pediatric centers, potentially overrepresenting severe disease while undercapturing mild or single-system *CFTR*-related disorders. Long-term follow-up is limited, and data on lung function trajectories, survival, transplantation, and late outcomes (including malignancy risk) remain sparse [6,8,9,11]; functional validation is also uneven: although splicing, protein expression, and channel function have been examined for selected variants [13,16,20,21,22,23,25,27,39], many variants are still classified primarily through inference rather than direct functional evidence. Treatment data are particularly limited, with few systematic reports on modulator efficacy and safety in Chinese patients.

### 6.2. Key Scientific Questions for Future Research

Based on current evidence, several priorities emerge: (1) Establish a national CF registry and pilot newborn screening: integrate genotype, phenotype, and outcome data across pediatric and adult patients; pilot CF inclusion in newborn screening in major cities or high-risk regions to assess feasibility and cost-effectiveness [7,9,11]. (2) Build systematic functional and drug-response pipelines for China-enriched variants: perform in vitro functional analyses and ex vivo theratyping for p.Gly970Asp, p.Ile1023Arg, p.Arg553Ter, and splicing/complex deletion genotypes; assess rescue potential using existing modulators to support variant-specific prescribing [13,16,18,19,20,21,39]. (3) *CFTR*-associated disease spectrum and pathological processes in the “CF-like” phase: Deepen understanding of the *CFTR*-associated disease spectrum, including CBAVD, recurrent pancreatitis, pseudo-Bartter syndrome-like electrolyte disturbances, and CF-associated liver disease, clarifying their continuum with classic CF [8,9,10,11,12,15,18,29,38,50]. Investigate the risk and mechanisms of progression to typical CF in these populations under the influence of environmental factors and comorbidities. (4) Interrogate CFTR’s roles in inflammation, tumorigenesis, and tissue repair: map network interactions involving CFTR–β-catenin, NF-κB, autophagy, and vascular responses; clarify bidirectional effects in tumor biology, vascular pathology, and musculoskeletal repair to enable cross-disease therapeutic hypotheses [22,23,24,25,26,27,28,29,30,31,32,33]. (5) Advance gene therapy and nucleic acid therapeutics relevant to China: design mutation-informed supplementation or editing strategies aligned with the local spectrum and develop ethically and regulatorily sound trial pathways for patients lacking effective pharmacologic options [45,46,66]. (6) Regional environment–genotype interactions: examine how geographically patterned environmental determinants (e.g., air quality, locally prevalent pathogens) interact with *CFTR* variants that are more specific to the Chinese population to shape disease phenotypes—for instance, evaluating the contribution of air pollution or regionally endemic infections to the progression of pulmonary lesions in Chinese patients with CF.

## 7. Summary and Prospect

Differences in *CFTR* mutation frequencies may be shaped by multiple evolutionary forces, including genetic drift, migration history, and natural selection. Regional variation in population genetic structure and selective pressures can increase the prevalence of specific variants in certain groups, thereby contributing to disparities in *CFTR* mutation frequencies and disease incidence across populations. CF in China is not “exceedingly rare” but rather “substantially underrecognized.” Epidemiological estimates indicate that although incidence is lower than in European-ancestry populations, it is meaningfully higher than suggested by current clinical reporting [7]. Contributing factors include limited clinical awareness, lack of newborn screening, and restricted access to genetic testing [6,8,9,10,11]. The mutation spectrum of CF in China is highly distinctive; p.Gly970Asp, p.Ile1023Arg, p.Arg553Ter, and others show significant enrichment in the Chinese population [7,9,11,14,15,16,18,20,21]. Numerous splicing variants and complex rearrangements further expand phenotypic diversity [13,15,17,18,19,20,39]. This genetic heterogeneity contributes to a broad phenotypic continuum, ranging from classic pediatric CF to *CFTR*-related disorders dominated by CBAVD, pseudo-Bartter syndrome-like electrolyte abnormalities, CF-associated liver disease, and recurrent pancreatitis [6,8,9,10,11,12,15,29,38,50]. These patterns support a shift from a single-disease framing toward a continuum model of *CFTR*-associated disease. Mechanistically, CFTR functions not only as an anion channel but also as a node within multisystem signaling networks, including Wnt/β-catenin, autophagy, NF-κB, vasoregulatory pathways, and tumor-associated signaling [22,23,24,25,26,27,28,29,30,31,32,33,37]. This not only explains the multi-organ involvement in CF patients but also provides a theoretical foundation for cross-disease targeted therapies. Precision medicine and gene therapy hold immense potential for the Chinese population, with gene supplementation, mRNA therapies, and gene editing offering hope for those without existing treatment options [45,46,66]. However, precision medicine does not resolve all scientific questions surrounding CF and faces multiple challenges: First, significant therapeutic heterogeneity persists. Second, a subset of patients (e.g., those with minimal function/truncated mutations or rare variants) remain unresponsive to existing modulators. Current CFTR modulators are primarily designed for p.Phe508del and specific mutations, offering limited coverage for Chinese patients [5,7,9,11,12,14,15,16,18,20,21]. Third, nonlinear interactions exist between the inflammatory microenvironment and drug responses. Fourth, while nucleic acid and gene therapies hold great promise, delivery barriers and long-term persistence remain critical bottlenecks [64,66].

Advancing these strategies in China will require coordinated efforts to address several persistent challenges, including incomplete functional characterization of *CFTR* variants, limited drug affordability and access, uneven clinical infrastructure, and insufficient capacity for long-term follow-up. In recent years, China has taken proactive steps to strengthen rare-disease diagnosis and treatment networks, streamline drug review pathways, and expand medical insurance coverage, thereby creating a more supportive institutional environment for managing rare genetic disorders such as CF. Looking ahead, expansion of multicenter collaborative cohorts, newborn screening pilot programs, and gene-therapy clinical trials may enable China to shift from a peripheral participant to a substantive contributor to global CF research, leveraging its large population and distinctive mutation spectrum.

For clinicians and translational investigators in China, presentations such as unexplained bronchiectasis or chronic cough accompanied by malnutrition or steatorrhea, idiopathic pseudo-Bartter syndrome, or male infertility due to CBAVD should raise suspicion for *CFTR*-related disorders and prompt timely genetic testing. Because *CFTR* variants differ markedly in functional consequences and clinical phenotypes, the Chinese mutation spectrum necessitates population-specific functional annotation and drug-response databases rather than direct extrapolation from Western cohorts. Beyond CFTR modulators, emerging approaches—including gene supplementation, mRNA-based therapy, and genome editing—are likely to first benefit patients with non-p.Phe508del variants for whom no approved targeted therapies exist. This unmet need represents both a major scientific challenge and a significant opportunity for innovation.

CF therapy is evolving from “repairing consequences” to “correcting root causes,” yet its ultimate goal should extend beyond stronger modulators. Based on this review, the next phase’s directions can be summarized as follows: utilizing whole-genome sequencing to guide patients into treatable pathways; expanding variant coverage and elevate efficacy ceilings through alternative pathways; re-evaluating the net benefits of inflammation and anti-inflammatory strategies in the “modulator era”; consolidating efficacy and safety through randomized trials and real-world clinical data; advancing mutation-agnostic therapies like inhaled mRNA while resolving engineering bottlenecks; and enabling computable drug iteration through structure-guided design. For China and other regions with highly dispersed mutation spectra, establishing local data and functional platforms is essential for achieving equitable precision medicine. Finally, we advocate for the early inclusion of CF in newborn screening programs to lower the rate of missed diagnosis, enable timely diagnosis and treatment, and strengthen the introduction and reimbursement support for rare-disease medicines. These efforts would improve access in China to innovative therapies such as CFTR modulators and ultimately lead to better patient outcomes.

## 8. Materials and Methods

A literature search was conducted via the PubMed database, covering the time period from 2015 to 2026 (encompassing all publicly available relevant publications). Search terms include “cystic fibrosis” or “Chinese people” with “cystic fibrosis” or “treatment methods” with “cystic fibrosis” or “modulators” with “cystic fibrosis”. Inclusion criteria: Research topics must pertain to CF, with a focus on clinical or genetic studies involving Chinese patients, or CF treatment methods and recent advances. Eligible literature types include case reports, case series, cohort studies, clinical trials, and relevant reviews. Cases must have a confirmed diagnosis (based on sweat chloride testing and/or *CFTR* gene testing). Exclusion Criteria: Research unrelated to cystic fibrosis or not involving the Chinese population/treatment strategies; studies lacking original data support or representing duplicate publications. Literature screening was conducted independently by two researchers: each initially screened the retrieved titles and abstracts to establish a list of studies meeting the inclusion criteria; subsequently, each independently assessed the full texts of the candidate studies. Any discrepancies arising during the screening process were resolved through discussion between the researchers. The initial literature screening yielded approximately 300 records. Following title and abstract screening, around 220 studies failing to meet the criteria were excluded. The remaining 80 studies underwent full-text evaluation. After further selection and exclusion, a total of 67 studies meeting the inclusion criteria were ultimately incorporated, providing further support for the conclusions of this research.

## Data Availability

No new data were created or analyzed in this study. Data sharing is not applicable to this article.

## Abbreviations

The following abbreviations are used in this manuscript:

AAV	adeno-associated virus
ABC	ATP-binding cassette
ASL	airway surface liquid
CBAVD	Congenital bilateral absence of the vas deferens
CF	Cystic fibrosis
CFTR	Cystic fibrosis transmembrane conductance regulator
CNVs	copy-number variants
ER	Endoplasmic reticulum
ETI	Elexacaftor/Tezacaftor/Ivacaftor
MSD	Membrane-spanning domain
MLPA	multiplex ligation-dependent probe amplification
NBD	Nucleotide-binding domain
NF-κB	Nuclear factor kappa-B
NRDL	National Reimbursement Drug List
R domain	Regulatory domain
WES	whole-exome sequencing
